# Population-level body condition correlates with productivity in an arctic wader, the dunlin *Calidris alpina*, during post-breeding migration

**DOI:** 10.1371/journal.pone.0187370

**Published:** 2017-11-01

**Authors:** Grzegorz Neubauer, Lucyna Pilacka, Piotr Zieliński, Jadwiga Gromadzka

**Affiliations:** 1 Ornithological Station, Museum and Institute of Zoology, Polish Academy of Sciences, Nadwiślańska 108, Gdańsk, Poland; 2 Laboratory of Forest Biology, Wrocław University, ul. Sienkiewicza 21, Wrocław, Poland; Hawaii Pacific University, UNITED STATES

## Abstract

Weather and predation constitute the two main factors affecting the breeding success of those Arctic waders whose productivity is highly variable over the years. We tested whether reproductive success is associated with the post-breeding condition of adults, in which in ‘good’ years (with warm weather, plentiful food and low predation pressure) the condition of breeders and their productivity is high. To verify this hypothesis, we used a 10-year dataset comprising 20,792 dunlins *Calidris alpina*, trapped during migration at a stopover site on the southern Baltic Sea shore. Males were consistently in a slightly worse condition than females, likely due to male-biased parental investment in brood rearing. Annual productivity indices were positively correlated with the respective condition indices of breeders from the Eurasian Arctic, indicating that in ‘good’ years, despite great effort spent on reproduction, breeders leave the breeding grounds in better condition. The pattern did not hold for 1992: productivity was low, but the average condition of adults during migration was the highest noted over the decade. We suggest that the delayed effect of the Mount Pinatubo eruption in the Philippines in 1991, could be responsible for the unexpected high condition of Arctic breeders in 1992. High population-level average condition, coupled with the low productivity could stem from severe weather caused by the volcano eruption a year before and strong predation pressure, which in turn lead to a reduced investment in reproduction. The importance of large-scale episodic phenomena, like this volcano eruption, may blur the statistical associations of wildlife with local environmental drivers.

## Introduction

Reproduction constitutes one of the biggest energetic efforts in birds’ annual cycle [1]. Arrival time, body condition at arrival [2, 3, 4, 5], along with local food availability [6, 7] and predation pressure [8, 9, 10, 11] shape the breeding output of avian species. In contrast to temperate, subtropical and tropical climatic zones, breeding conditions remain unpredictable in species breeding in the Arctic. This is due to strong annual fluctuations of the two main factors limiting breeding success–predation pressure and food availability, both affected to a large degree by weather [12, 13]. Consequently, Arctic breeders, including many wader species, experience variable breeding success in response to conditions in a given year. Due to their small to medium size, waders are ‘income breeders’, unable to both complete migration and produce eggs solely using energy stores accumulated at stopover sites—to start reproduction they must feed after arrival at the breeding grounds [3, 14]. The subsequent laying period is energy intensive due to large–in relation to body size–eggs produced by waders and other activities performed at the same time, such as mating and site defence. Thereafter birds bear the high costs of chick rearing and nest defence [13]. In addition, a short breeding period, resulting from a seasonal food peak, defines the optimal time window for reproduction. With such constraints, an early arrival might be favourable, but it encompasses the risk that birds might face the problem of limited food supply due to frozen ground and persisting snow cover [5]. This may lead to a reduction in reproductive output or even complete breeding failures. In extreme cases with severe weather conditions birds can skip breeding and undertake reverse migration [12].

The body condition of birds during migration, at their arrival at the breeding grounds, during egg-laying and chick rearing periods has been well studied [5, 6, 15, 16, 17, 18]. We might expect that following the energy-consuming breeding, the body condition of breeders may be poor. On the other hand, in favourable years, with both high food availability and low predation pressure, birds may leave the breeding grounds in good shape. However, according to our best knowledge there are no previous studies documenting the relationship between the annual post-breeding body condition and reproductive success. In this study, we focus on investigating this relationship, using the dunlin *Calidris alpina* as an example.

Despite both sexes performing brood care in dunlin, there is a male-biased effort in chick rearing. Females are fully involved only at the incubation stage (which is believed to be less costly than chick rearing [19, 20]) and desert broods soon–up to 12 days–after hatching [21, 22, 23]. In consequence, males bear higher costs of reproduction than females and their body condition after breeding might be expected to be lower than that of females [24].

Most Calidrid species do not attempt breeding when one year old (their second year of life) and they generally stay at the wintering grounds or nearby. The dunlin represents an exception: birds in their second year of life (hereafter ‘immatures’) undertake northward migration and attempt to breed [15, 25, 26]. A first breeding attempt can be more energetically demanding than in older birds, due both to inexperience in breeding site selection (which may lead to breeding at nest sites with low food abundance and high predation risk) and occupancy of better quality sites by older birds (intraspecific competition). Moreover, immature dunlins face stronger time pressures as they arrive at the breeding grounds later [21]. It has been well documented that early arrival at the breeding grounds may be selected for in arctic waders and positively influences breeding success [13, 27, 28, 29]. Immatures usually leave on their southward migration earlier than adults [30, 31], which might also influence their post-breeding condition.

Three out of nine recognized dunlin subspecies [32] occur at the Vistula mouth stopover site: *alpina*, *centralis* and *schinzii* [33, 34, 35]. They breed in different geographic regions: *alpina* occupies the western Eurasian Arctic, while *centralis* breeds in the more eastern part of the range. The *schinzii* subspecies’ breeding range is restricted to lower latitudes, mostly around the Baltic Sea [32]. Migration of the two high-Arctic subspecies differ in the distances to be traversed and in the duration of their migration. The migration routes of these subspecies overlap in birds which migrate west and then south-west to south to reach wintering grounds in southern and western Europe and northern Africa [32]. Therefore, they face different constraints during the breeding and migration periods and differences in the post-breeding body condition of birds from different origins can be expected to vary accordingly: eastern birds during their migration through Europe might be in worse condition due to their longer journey.

The proportion of first-year birds recorded during migration or at the winter quarters represents an approximate measure of breeding success in a given year [30, 36]. In various wader species, the annual proportion of juveniles among all individuals of a given species, can vary between 0.1 and 0.8, indicating good or poor breeding seasons [9, 37, 38]. Because of both (1) strong annual variation in productivity and (2) the ease of obtaining productivity indices with census and monitoring programmes on multiple species [39, 40], waders constitute an excellent group to test various predictions related to energy expenditure, breeding success and the resulting body condition. In Arctic waders, their pre-breeding body condition varies in response to their migratory strategy [41, 42], age [33, 43] and arrival stores which are known to affect breeding success [5]. However, the relationship between the reproductive success and the post-breeding condition remains unexplored. Costs of reproduction affect fitness [44, 45] and successful reproduction might turn out to be costly immediately: high energy expenditure may lead to a negative relationship between indices of productivity and post-breeding condition of breeders. On the other hand, in years with plentiful food and low predation, both productivity and the condition of breeders might be high. Given both that waders are commonly studied birds and the availability of large datasets on multiple species, it remains surprising that a relationship between yearly indices of productivity and the post-breeding condition of breeders has not yet been addressed.

In the present work, we investigate this relationship in the dunlin, a long-distance migrant, wader species breeding in the high Arctic. We used a 10-year dataset of dunlins, trapped at the stopover site during their southward migration, to test if the condition of breeders correlates with productivity. Additionally, we attempt to identify factors responsible for the variation in condition, by answering the following questions: (i) do males have a poorer post-breeding condition, due to their higher investment in raising broods; (ii) are immatures in worse condition compared to older birds due to being unexperienced; and (iii) is there any effect of origin–i.e., do ‘western’ and ‘eastern’ birds differ in condition, in line with the distance passed during migration.

## Material and methods

We used data of migrant dunlins trapped at the stopover site in the Vistula Mouth, Poland (54°22’N, 18°57’E) between 1990 and 2000. The year 1997 was excluded because a flood during the migration peak in July resulted in most birds being missed, as trapping had to cease. Birds were stopping over to feed on flat, sandy beaches and annually trapped with walk-in traps between the 12^th^ July and 25^th^ September (exact dates of the beginning and ending of trapping differed by 1–3 days). Birds were removed from the traps by professional ornithologists and volunteers at two-hour intervals each day (from 05:00 to 23:00 CET) and transported to the ringing station located outside the feeding areas, at a distance of up to some hundred meters from the trapping area. Each bird was measured, weighted, ringed and released immediately after processing. A set of measurements were taken, including body mass (to the nearest 1 g), wing length (ruler, to the nearest 1 mm), tarsus length (calliper, to the nearest 0.1 mm) and bill length (calliper, to the nearest 0.1 mm). All these measurements showed some sexual dimorphism [46], but we have chosen the bill length as a measure of body size (see below). Individuals were aged according to plumage characteristics: adults by breeding plumage, its remnants or the colour of inner median coverts ([47], following [48], see also [49]). Immatures were identified by the presence of worn, juvenile inner median coverts. We also classified each individual as originating from one of the two regions of the Arctic (hereafter ‘western’ or ‘eastern’). This distinction was made on the presence of so-called ‘adult-buff’ coverts, as their presence indicates eastern origin [33, 49, 50]. Juveniles were identified by plumage characters [47, 49, 51]–a lack of black (adult type) patch on the belly, buffish edges of inner median coverts and tertials and freshness of the plumage; no wear scores had to be used. Adult birds were sexed by a discriminant function [46]. Juveniles could not be reliably sexed since their bills and wings might not be fully-grown during the first migration. Following the above classification, eight age-sex-origin groups were defined for non-juvenile birds. The proportion of juveniles among all trapped individuals in a given year was used as the estimate of productivity, which is believed to be a reliable index of breeding success and allows to separate ‘good’ from ‘poor’ years [52, 53]. In total, 20,792 individuals were trapped, measured and ringed over the study period, including 5,171 adult males, 2,747 adult females, 2,346 immature males, 1,185 immature females and 9,144 juveniles. A male-biased sex ratio was evident in non-juveniles (immatures– 68.5%, adults– 65.7%) and is well known in dunlin at the chicks stage (61,1%, [54]), at the stopover during migration (immatures– 60,3%, adults– 59,4%, [55]) and at various winter quarters (Portugal– 75% [56], Pacific Coast– 61% and 65% [57]). The measurement data were carefully checked prior to analysis and all individuals with missing measurements, and a further 17 birds with extreme measurements, most probably due to observer errors, were excluded. We divided our analysis into two steps: first, we focused on estimating the population-level, mean annual condition indices of migrants; secondly, we assessed how these estimates relate to productivity indices. Although it is possible to directly assess the effect of productivity on condition by including the former as a predictor of condition in the model, we preferred a more conservative, although less powerful, approach: condition indices were estimated for a given year, origin, sex and age groups, but were not modelled as a function of productivity. In this approach, the first analysis aimed at estimating the yearly mean condition index, along with 95% confidence intervals for each group, and included only the first captures of the same birds in a given year (as some were trapped several times during their stay) and only non-juvenile (i.e., potential breeders) individuals (n = 11,432, Table 1).

**Table 1:** Yearly numbers of non-juvenile dunlins (adults, immatures: 2nd calendar year) trapped at the Vistula Mouth during southward migration in relation to origin, sex and age. The total number of captured juveniles per year is also shown.

Group	Year										
	1990	1991	1992	1993	1994	1995	1996	1998	1999	2000	total
‘western’ birds											
adult females	225	393	331	168	216	116	280	321	152	272	2474
adult males	551	701	448	419	347	233	450	573	285	551	4558
immature females	18	229	111	21	138	35	77	93	24	219	965
immature males	69	306	290	33	224	80	190	154	52	313	1711
‘eastern’ birds											
adult females	28	57	52	14	30	22	7	25	16	16	267
adult males	1	46	68	2	38	15	20	11	3	15	219
immature females	60	90	111	60	39	78	33	45	46	45	607
immature males	9	84	173	10	100	83	55	48	2	67	631
juveniles	1932	2405	399	2199	434	377	164	430	569	434	9343
total	2893	4311	1983	2926	1566	1039	1276	1700	1149	1932	20775

Because body mass is size-dependent, we used residual body mass from regression of body mass on bill length as an index of body condition at the individual level. This index is commonly used in waders [15, 58], while using wing length is not recommended due to wear and moult [58]. Tarsus length can also be used, as it is significantly correlated to bill length (in non-juveniles: r = 0.70, P < 0.001, df = 11176), and these two traits are similarly informative in respect to body size. In our dataset, correlations between the residual body mass of non-juveniles calculated using bill and tarsus lengths were strongly correlated for any single year (>0.92, which means these two variables share ~80% of variability).

We used additive linear mixed effects models (GAMMs) with normal error distribution for their flexibility in capturing non-linear patterns via smoothers and where the optimal amount of smoothing is obtained via cross-validation [59, 60]. The fixed factors included sex, age, origin and their interactions. Variation due to year was allowed to differ between birds of ‘western’ and ‘eastern’ origin and was addressed with the two smoothers, estimated separately for the origin groups. Alternatively, models with just a single smoother, common to both origin groups and the remaining fixed effects as described above, were also fitted, reflecting the hypothesis that the annual condition varies similarly in ‘western’ and ‘eastern’ dunlins. A year-group combination factor was included as a random effect (80 levels) to control for non-independence (correlated condition indices) among birds of the same age, sex and origin in a given year. To address correlated condition indices in subsequent years (inter-annual non-independence) we accounted for this temporal autocorrelation by incorporating the autocorrelation structure into the models. Keeping the systematic and random parts as in the global model, we fitted models with nine auto-regressive moving average (ARMA) correlation structures, differing in the number of estimated parameters. A correlation with a single auto-regressive parameter and a single moving-average parameter was found to be the best-supported by AIC, though two other were nearly equally good (a difference of 2.34 and 3.54 AIC units). We applied the best-supported structure in all the models; this choice is further supported by the fact that models with the same fixed and random parts and different correlation structures produced nearly identical results in terms of parameter estimates, *t*-statistics, *P*-values and predictions (in line with [61]).

We first fitted the global model with age, sex, origin and their interactions as fixed factors and the two smoothers (one estimated for ‘western’ and another one for ‘eastern’ birds). After fitting the global model, using the mgcv R-Package [62], we proceeded with fitting simpler, nested models with all the remaining combinations of explanatory variables manually. 28 models were fitted in total, including 14 models with the two smoothers, 14 with a single smoother and two ‘null’ models with smoothers only (one or two) and no other explanatory variables; ‘true null’ models, with no smoothers, were not considered. Model ranking was based on AIC value [63] and because it was relatively balanced, model-averaging was necessary. We refitted models in the MuMIn [64] library to get model-averaged parameter estimates (i.e., considering model weights). Model-averaged predicted condition for a given year and group were obtained manually since smoothers in GAM/GAMM models are not supported by prediction functions after model-averaging has been applied [65]. Twelve out of 28 models (with Δ AIC ≤ 10 and ω AIC > 0.005) were taken into account to calculate the model-averaged predictions presented.

## Results

**Fig 1:**
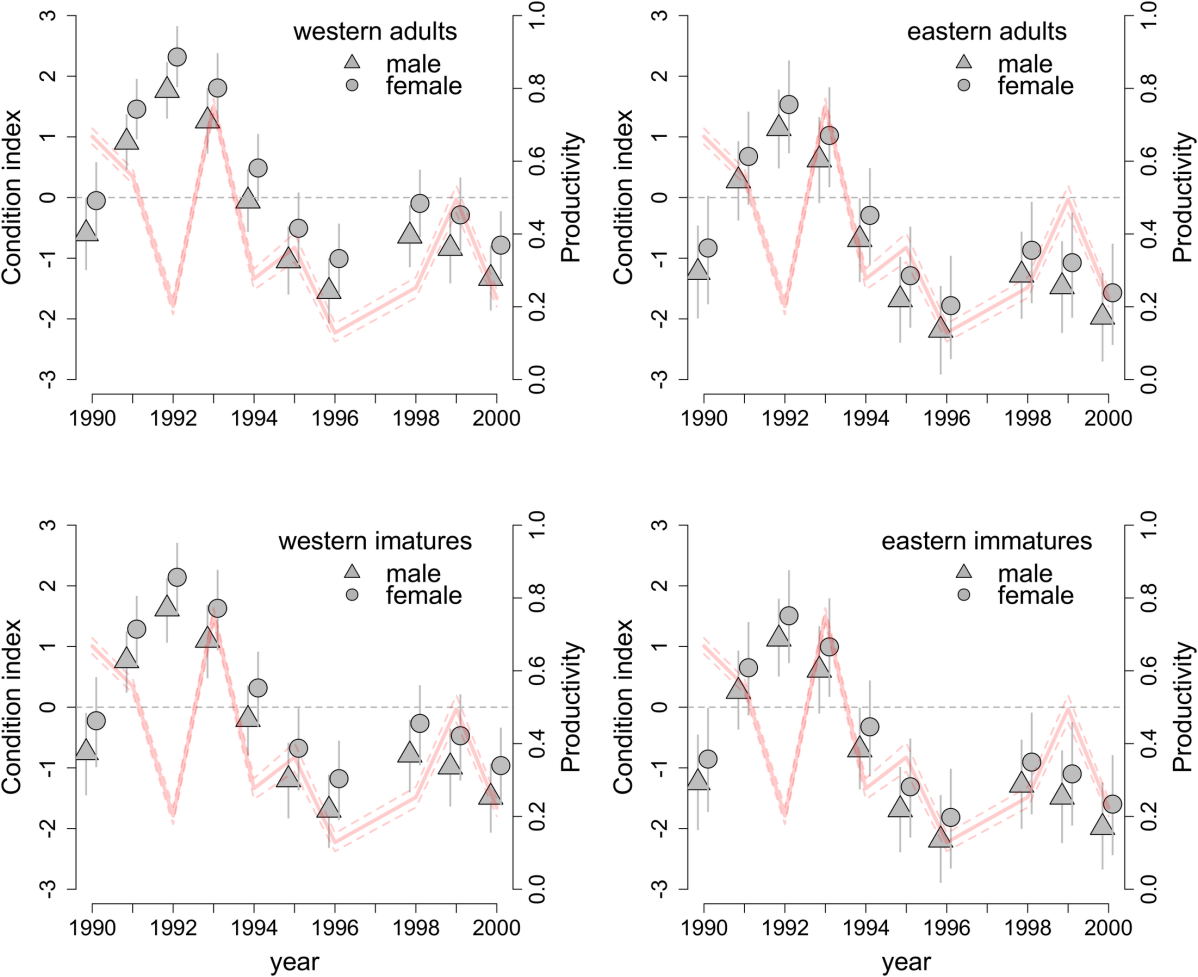
Annual post-breeding body condition indices of migrant dunlins in relation to age, sex and origin. Grey symbols represent mean condition indices for a given group in subsequent years and whiskers indicate 95% confidence intervals. Pale red lines show productivity indices (solid) with 95% confidence intervals (dashed). The dashed grey horizontal line denotes zero, which approximates the mean condition index of all non-juveniles across all years.

Across the entire dataset, in non-juveniles, the mean yearly body condition varied significantly over the years (Fig 1), with the lowest mean for 1996 (–1.74), the highest for 1992 (1.87) and the grand mean very close to zero (mean –1.33e-15 ± 4.01 SD, median –0.66, 95% CI: –6.10 to 9.61). The wide min-max range for any given year indicated the presence of birds in very poor and very good condition regardless of year. The top four models (ω AIC between 0.20 and 0.24) included a single smoother, and fixed sex, origin and (in two models) age effects, with interactions between sex and origin or age and origin (Table 2). Further models with some support were similar in that they included a single smoother and either missed various fixed effects or did not (Table 2). The best-supported model with two smoothers had AIC weight < 0.0001, implying that interannual variation in condition is similar in both origin groups and the patterns are sufficiently well approximated by a single smoother at lower statistical cost (Table 2). Indeed, when origin groups were modelled with two smoothers, the patterns were similar. In the best-supported four models, the high degree of non-linearity of a smoother was nearly identical (effective degrees of freedom, *edf*, between 6.31 and 6.33, S1 Fig) and confirmed highly variable condition across years, reflecting the pattern seen in the raw data.

**Table 2:** GAMMs used to explain variance in body condition indices of dunlins trapped at the Vistula mouth, Poland, during southward migration periods between 1990 and 2000. The number in the column entitled ‘smoothers’ indicates whether a model includes a single or two smoothing functions, ‘×’ indicates interaction between fixed factors. All the models included the random effect and the auto-correlation structure (see Methods). The best-supported model is given in italics. Models 1–12 were used in model-averaging.

Model	Fixed factors	smoothers	AIC	Δ AIC	ω AIC
*1*	*sex + origin*	*1*	*62204*.*24*	*0*.*00*	*0*.*239*
2	sex × origin	1	62204.50	0.26	0.210
3	sex + age × origin	1	62204.65	0.41	0.195
4	sex + age + origin	1	62204.65	0.41	0.195
5	sex × age + origin	1	62206.32	2.08	0.084
6	sex × age × origin	1	62208.74	4.50	0.025
7	origin	1	62210.30	6.06	0.012
8	sex + age	1	62210.39	6.15	0.011
9	sex + origin	1	62210.84	6.60	0.009
10	sex × origin	1	62211.17	6.93	0.007
11	sex	1	62211.20	6.96	0.007
12	sex × age	1	62212.16	7.92	0.005
13	age	1	62216.38	12.14	0.001

The pattern across years was similar in both ‘western’ and ‘eastern’ birds, with the best condition in 1992–1993 and the poorest in 1996 (Fig 1). Males had a significantly poorer condition than females in all the years (Table 3). Similarly, irrespective of sex and age, ‘eastern’ birds were in significantly worse condition than ‘western’ birds (Table 3, Fig 1). Both differences were not large, but significant.

**Table 3:** Model-averaged coefficient estimates from the best-supported GAMMs explaining variation in condition indices of migrant dunlins. Models with Δ AIC ≤ 10 were considered to produce model-averaged coefficients.

Coefficient	Estimate	S.E.	z	P
Intercept	0.508	0.184	2.762	0.006
origin: 'eastern'	-0.897	0.406	2.209	0.027
sex: male	-0.579	0.217	2.666	0.008
origin: ‘eastern’ x sex: male	0.277	0.433	0.640	0.522
age: immature	-0.241	0.264	0.911	0.362
origin: 'eastern' x age: immature	0.216	0.401	0.539	0.590
age: immature x sex: male	0.038	0.192	0.198	0.843
origin: ‘eastern’ x age: immature x sex: male	-0.003	0.120	0.029	0.977

**Fig 2:**
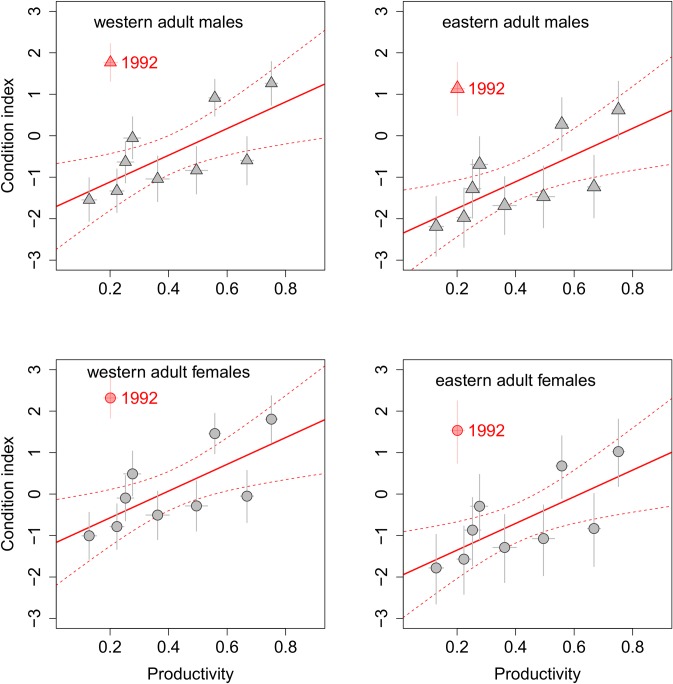
Annual post-breeding body condition indices of adult dunlins plotted against productivity indices in 1990–2000 (excluding 1997). The outlying year 1992 is marked with red. Symbols represent mean productivity and mean condition index for one year, error bars– 95% confidence intervals for both productivity and condition indices. Regression lines (bold–mean, dashed– 95% confidence intervals) are drawn to illustrate the relationship for nine years, after excluding 1992. The relationship for immatures was nearly identical (see S2 Fig).

Yearly productivity indices varied strongly, from 0.12 in 1996 to 0.75 in 1993 (Fig 1). Estimated mean yearly body condition indices showed strong and significant positive correlations with productivity for all groups after excluding the outstanding 1992 year. All the correlations were around 0.72 (‘western’ birds: adult males: r = 0.716, P = 0.029, adult females, r = 0.718, P = 0.030, immature males: r = 0.715, P = 0.030, immature females: r = 0.716, P = 0.030; ‘eastern’ birds: adult males: r = 0.717, P = 0.029, adult females: r = 0.716, P = 0.03, immature males: r = 0.716, P = 0.030, immature females: r = 0.718, P = 0.029, all df = 7; Fig 2, S2 Fig).

1992 was the outlying year (Z scores 4.26–4.28, depending on the group), with the best condition (estimated group means between 1.31 and 2.12) and the second-lowest productivity (0.20) over the decade, implying that the pattern observed for the whole period does not hold (Fig 2).

## Discussion

### Variation in body condition—The influence of sex, age and origin

There is a great linkage between all stages of the annual cycle, especially in migratory animals. Recent ecological studies recognize this phenomenon as carry-over effects: events that occur in one season (or stage), influence fitness at both the individual and population level in the following season (or stage) [66, 67]. This concept may explain the relationship between breeding success, feeding conditions at the stopover site, wintering grounds quality and body condition in a given year [68, 69, 70, 71, 72, 73, 74]. Our study did not assume to describe carry-over effects but we are aware that body condition at each stage of the annual cycle can be influenced by an individual’s history and performance at the previous stages. Body condition during the post-breeding period might be affected by the reproductive effort in a given breeding season, stemming from feeding conditions and predation pressure at the breeding grounds, as well as by the habitat quality (i.e., food availability) at stopover sites visited during the southward migration. Sex, age, and origin influence both breeding and migration strategies, which in turn are linked to variation in body condition.

Our results confirm the assumption that male-biased effort in chick rearing period affects the males’ body condition in dunlin. The differences noted were not large, but consistent and highly significant. In all years, males were in poorer condition than females, irrespective of age and origin. Despite the fact that females incur the undoubtedly high costs of egg laying, males apparently face higher energy expenditures resulting from nest and chicks’ defence after female desertion.

We found it surprising that there was little support for the age effect. Immature dunlins undertake breeding in their second year of life [15, 25, 26] and due to their low experience and late arrival to the breeding grounds [21] might bear higher costs of this attempt. Apparently, in our dataset, the signal for the age effects on condition was too weak to gain substantial support.

Dunlin subspecies overlap in morphological characters, which makes it difficult to determine the origin of migrant birds observed at stopover sites and wintering grounds [34, 75]. There are only a few ringing recoveries between the Vistula Mouth stopover site and the breeding grounds, indicating that dunlins migrating through the Baltic region originate from the Eurasian Arctic, including Siberia [76, 77]. At the same time, it is known that birds from the easternmost part of the nominate subspecies *alpina* breeding range (east from Yamal Peninsula) annually migrate through the region in lower numbers than their western counterparts [33, 35, 77]. The western limit of Siberian subspecies *centralis* breeding range remains unclear [78, 79] and genetic studies suggest the persistence of *alpina/centralis* contact zone [80], but still, the occurrence of westernmost *centralis* at the southern Baltic during southward migration is likely [33, 34, 35]. Mitochondrial DNA analyses confirm both Siberian and European dunlin haplotypes were found at the Vistula Mouth stopover site [34, 75, 81]. Therefore, our conclusions cannot be directly applied to any given population or area, apart from the rather rough distinction based on plumage (‘western’ vs ‘eastern’ birds) we have made. The presence of the ‘adult buff’ inner median coverts indicates the eastern origin of dunlins occurring each year at the Polish stopover site. Based on the analysis of museum specimens, it has been confirmed that the presence of the ‘adult buff’ type coverts is associated with birds breeding mainly in Eastern and Central Siberia [33, 79]. It means that birds captured at the Vistula mouth possessing ‘adult buff’ type coverts (treated here as ‘eastern’ birds), may originate either from the easternmost *alpina* range or may represent westernmost *centralis*. ‘Western’ dunlins are either *alpina* or–to a minor extent, given its small and declining population—*schinzii*. The difference in body condition observed between ‘eastern’ and ‘western’ birds may thus be a consequence of the difference in the distance covered between their breeding and wintering grounds. Our results indicate that ‘eastern’ birds had worse body condition than their ‘western’ counterparts. Dunlins breeding further east cover longer distances during their southward migration [34]. For this reason, ‘eastern’ birds might migrate under stronger time pressures and perform on average longer flights between subsequent stopover sites.

### The relationship between body condition and productivity

The results indicate that there is a strong positive relationship between the condition of dunlins during the post-breeding migration time and productivity in a given year. In years with higher productivity, the condition of breeders was also higher, with the exception of 1992.

Our findings suggest that in spite of the high energetic costs of reproduction, high breeding output may be achieved without significant reduction of adults’ body condition in favourable years. It has been well documented in waders that production of juveniles is strongly influenced by feeding conditions during the pre-fledging period [13] and summer temperature at the breeding grounds [38, 82]. If the feeding and weather conditions are favourable for chick development and survival, they are apparently sufficient for adults to keep in good condition as well.

A potential caveat that could limit our conclusions include the fact that some juveniles might migrate through the Baltic after the trapping period at our stopover site [83] and thus, our productivity indices might be underestimated. The migration dynamics varies from year to year, with an average 70% of birds passing during the study period, but if a large fraction of juveniles passed after the trapping was stopped, our estimates of productivity could be negatively biased for these years. Another question is how well trapping reflects juvenile proportions: most birds pass through the southern Baltic without stopping over at the Vistula mouth, and in general migration dynamics patterns based on trapping are known to differ from the ones based on visual observations at the same site [84]. We verified our productivity estimates by comparing them with the same annual estimates, but based on counts, obtained at another stopover site in the Gdańsk Bay–in the mouth of the Reda river, c. 42 km north-west from our study site [30]. We found that productivity indices obtained at both sites were significantly correlated (r = 0.71, P = 0.03, df = 8). At the same time, the estimates of productivity based on counts were highly (r = 0.81) correlated with estimates based on trapping results at the same site [30]. We therefore believe, that it is safe to assume that our productivity indices are reliable; at least, they similarly assess annual productivity as independently assessed at a nearby site. To conclude, the pattern seen over 10 years is unlikely to be a coincidence, and despite our study being fully correlative, we find the main results suggestive: ‘good’ years show both high productivity and good post-breeding condition of the breeding fraction of the population.

### The effect of extreme climatic events

Our dataset included the year 1992, when an aerosol cloud originating from the 1991 eruption of Mount Pinatubo in the Phillipines led to severe weather conditions and heavily affected avian reproduction over most of the Arctic [12]. In consequence of the eruption, aerosol clouds caused significant cooling in the spring and summer of 1992 over almost the entire Arctic. In 1992 spring was late, snow-melt delayed and summer started two-three weeks later than in average years, which caused the reduction of potential nesting area and food availability for waders [85]. Severe weather conditions resulted in a higher than usual proportion of non-breeders and a delay in breeding initiation in many species [12]. That the volcano eruption could also affect the condition of birds breeding in the Arctic seems logical, but it has not been shown so far. Our findings on migrant dunlins could be the first evidence in this respect: skipping breeding could lead to energy savings resulting in good body condition.

The unusually good condition coupled with low productivity in 1992, clearly outstanding from the overall pattern, could be caused by the cumulative effects of multiple factors. Low numbers of juvenile dunlins were also reported at other stopover sites in Europe, both during the autumn migration in 1992 and the spring migration in 1993 [52]. Probably, the best explanation of this phenomenon is the eruption of Mount Pinatubo in 1991. Waterbird species (waders and ducks primarily) constitute an alternative prey (mostly for Arctic foxes *Alopex lagopus*) in low rodent years and wader productivity in a given year is associated with the phase of the rodent cycle [85, 86, 87]. Irrespective of the bad weather conditions caused by the volcano eruption, the low abundance of rodents (lemmings and voles *Microtus sp*.) in 1992 and the high predation rate on birds’ clutches additionally affected Arctic-nesting waterfowl and the breeding success of waders (including dunlin) to a large degree (estimates of breeding success ranged from 0% to 10%) [85]. These effects in 1992 were spatially highly variable [12, 85] but did not mask the exceptionally low productivity in 1992. Therefore, the good body condition of dunlins trapped at the Baltic stopover site may suggest that they ceased nesting at an early stage or even didn’t attempt to breed, therefore saving energy that in normal conditions would be spent on breeding. This is further supported by the low productivity index of 1992 and corresponds with the findings of Ganter & Boyd [12], who reported a reverse migration of geese and waders (turnstones *Arenaria interpres*, grey phalaropes *Phalaropus fulicarius* and white-rumped sandpipers *Calidris fuscicollis*) in 1992. From the dunlin capture data at our study site (1990–2000) we conclude that southward migration in 1992 was on the same dates as in remaining years, indicating no earlier migration to winter quarters. Gunnarsson et al. [88] also reported that volcanic eruptions seem to have short-term but high-magnitude impact on the productivity of birds. Black-tailed godwit *Limosa limosa islandica* and whimbrel *Numenius phaeopus* productivity was reduced to almost zero after two volcanoes erupted in 2010 and 2011 in southern Iceland, but during the subsequent years productivity substantially increased. These findings suggest that unpredictable phenomena, which occur rarely, may significantly affect the performance of animal populations even at large distances–as in the case of the 1991 eruption. If missed, some patterns that hold may appear ‘insignificant’ or easily go undetected.

## Supporting information

S1 Fig Smoothing functions estimated by four best-supported models. (TIF)

S2 Fig Annual post-breeding body condition indices of immature dunlins plotted against productivity indices in 1990–2000 (excluding 1997). The outlying year 1992 is marked with red. Symbols represent mean productivity and mean condition index for one year, error bars– 95% confidence intervals for both productivity and condition indices. Regression lines (bold–mean, dashed– 95% confidence intervals) are drawn to illustrate relationship for nine years, after excluding 1992.
(TIF)

S1 File Dataset. (TXT)
