# Supplementary figures and images for: Population-level body condition correlates with productivity in an arctic wader, the dunlin Calidris alpina, during post-breeding migration
Source: PLoS One. 2017 Nov 1;12(11):e0187370. doi: 10.1371/journal.pone.0187370 (PMC5665542; doi:10.1371/journal.pone.0187370)

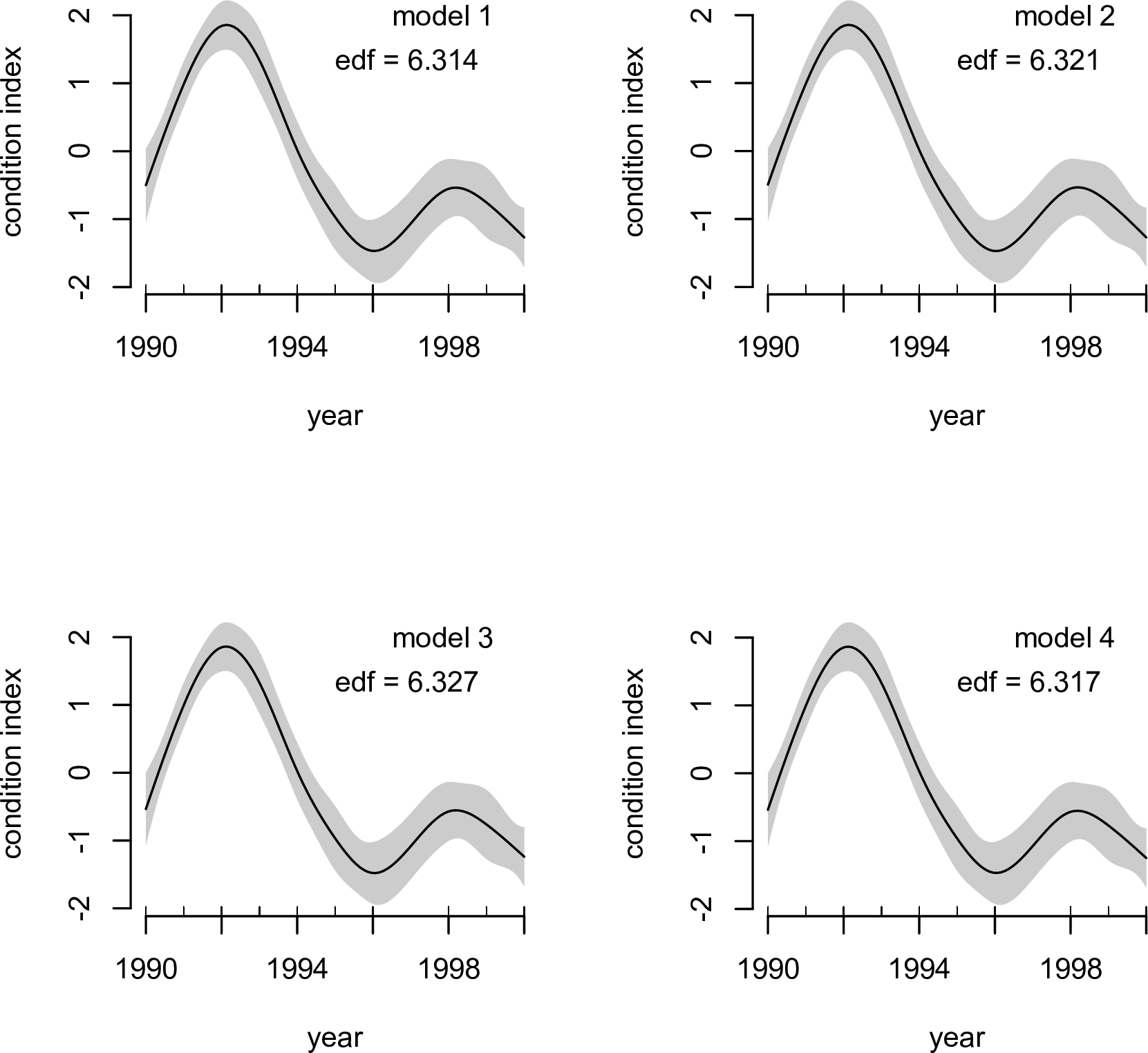

Supplement: S1 Fig — (TIF) [file pone.0187370.s001.tif]

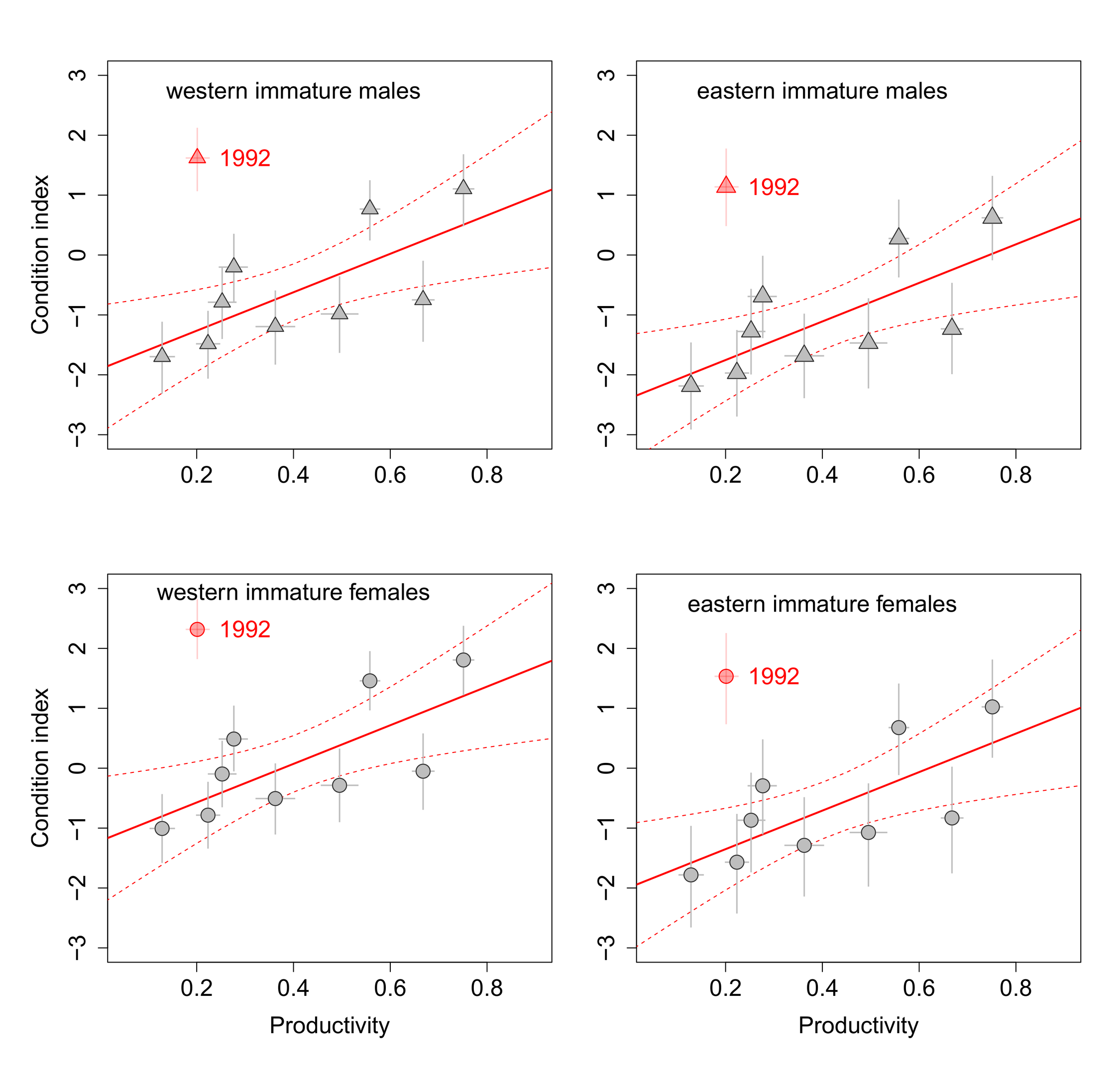

Supplement: S2 Fig — The outlying year 1992 is marked with red. Symbols represent mean productivity and mean condition index for one year, error bars– 95% confidence intervals for both productivity and condition indices. Regression lines (bold–mean, dashed– 95% confidence intervals) are drawn to illustrate relationship for nine years, after excluding 1992. (TIF) [file pone.0187370.s002.tif]
